# *trans*-5-(4-Chloro­phen­yl)*-N*-cyclo­hexyl-4-methyl-2-oxo-1,3-thia­zolidine-3-carboxamide

**DOI:** 10.1107/S1600536808012634

**Published:** 2008-05-07

**Authors:** Ying-Hui Yu, Ji-Long Liu, Li-Zhong Niu, Guang-Feng Hou, Jin-Sheng Gao

**Affiliations:** a College of Chemistry and Materials Science, Heilongjiang University, Harbin 150080, People’s Republic of China

## Abstract

The title pesticide, C_17_H_21_ClN_2_O_2_S, has a *trans* arrangement of the 4-chloro­phenyl and 4-methyl substituents of the thia­zolidine ring; the structure features an intra­molecular amide–ring carbonyl N—H⋯O hydrogen bond. The thia­zolidine ring is almost planar, the largest deviation being 0.199 (1) Å for the methyl-substitued C atom, and the cyclohexane ring has a chair conformation.

## Related literature

For the synthesis of the pesticide Hexythia­zox, see: Iwataki *et al.* (1981 ▶); Yamada *et al.* (1983 ▶). 
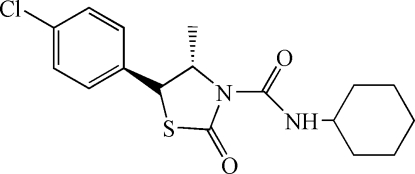


## Experimental

### 

#### Crystal data

C_17_H_21_ClN_2_O_2_S
*M* *_r_* = 352.88
Monoclinic, 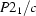

*a* = 10.284 (4) Å
*b* = 11.799 (5) Å
*c* = 15.902 (5) Å
β = 111.830 (14)°
*V* = 1791.2 (12) Å^3^
*Z* = 4
Mo *K*α radiation
μ = 0.34 mm^−1^
*T* = 291 (2) K
0.27 × 0.26 × 0.25 mm

#### Data collection

Rigaku R-AXIS RAPID diffractometer
Absorption correction: multi-scan (*ABSCOR*; Higashi, 1995 ▶) *T*_min_ = 0.913, *T*_max_ = 0.918
17194 measured reflections
4088 independent reflections
3066 reflections with *I* > 2σ(*I*)
*R*_int_ = 0.033

#### Refinement

*R*[*F* ^2^ > 2σ(*F* ^2^)] = 0.041
*wR*(*F* ^2^) = 0.109
*S* = 1.06
4088 reflections
209 parameters
H-atom parameters constrained
Δρ_max_ = 0.27 e Å^−3^
Δρ_min_ = −0.27 e Å^−3^

### 

Data collection: *RAPID-AUTO* (Rigaku, 1998 ▶); cell refinement: *RAPID-AUTO*; data reduction: *CrystalStructure* (Rigaku/MSC, 2002 ▶); program(s) used to solve structure: *SHELXS97* (Sheldrick, 2008 ▶); program(s) used to refine structure: *SHELXL97* (Sheldrick, 2008 ▶); molecular graphics: *SHELXTL* (Sheldrick, 2008 ▶); software used to prepare material for publication: *SHELXL97*.

## Supplementary Material

Crystal structure: contains datablocks global, I. DOI: 10.1107/S1600536808012634/ng2450sup1.cif

Structure factors: contains datablocks I. DOI: 10.1107/S1600536808012634/ng2450Isup2.hkl

Additional supplementary materials:  crystallographic information; 3D view; checkCIF report

## Figures and Tables

**Table 1:** Hydrogen-bond geometry (Å, °)

*D*—H⋯*A*	*D*—H	H⋯*A*	*D*⋯*A*	*D*—H⋯*A*
N2—H10⋯O1	0.84	2.03	2.706 (2)	137
C2—H1⋯O2^i^	0.93	2.47	3.386 (2)	170
C5—H3⋯S1	0.93	2.79	3.168 (2)	105
C12—H11⋯O2	0.98	2.44	2.831 (2)	103

Symmetry code: (i) 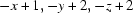.

**Fig. 1.**
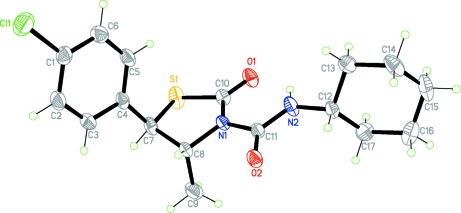
The molecular structure of (I), showing displacement ellipsoids at the 30% probability level for non-H atoms.

**Fig. 2.**
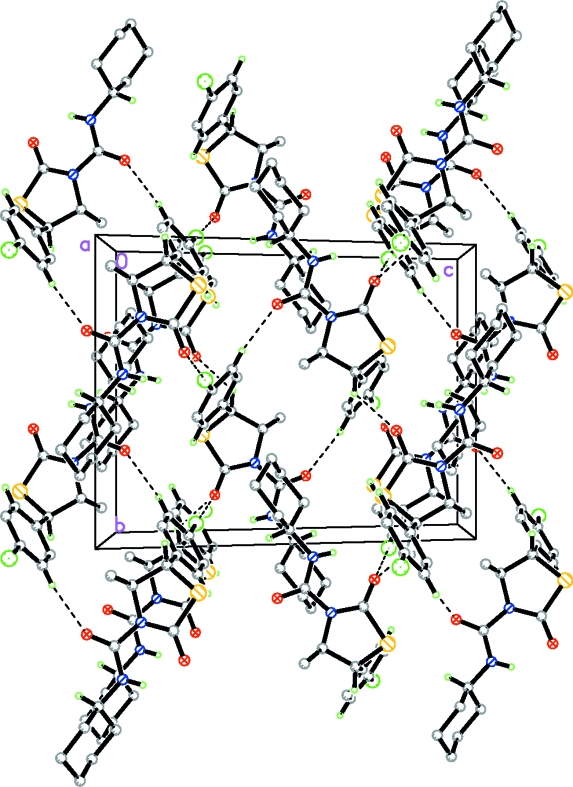
A partial packing view, showing the three-dimensional hydrogen-bonding network. Dashed lines indicate the hydrogen-bonding interactions. H atoms not involved in hydrogen bonds have been omitted for clarity.

## supplementary crystallographic information

### Comment

Hexythiazox, chemically named *trans*-5-(4-chlorophenyl)- N-cyclohexyl-4-methyl-2-oxo-3-thiazolidinecarboxamide, is known as a high efficiency of pesticide. In this paper, we first report the crystal structure of hexythiazox (I).

The title compound (I), consists of a planar phenyl ring (A), a S-contained five-numbers ring (B) and a cyclohexane ring (C). The S-contained five-numbers ring is alomst coplanar, with the largest deviation being 0.199 (1) Å for atom C8, and the cyclohexane ring is chair forms. The three rings make the following dihedral angles: A/B 82.20 (0.06)°, A/C 54.22 (0.07)° and B/C 81.70 (0.06)°.

In the crystal structure, an extensive network of intramolecular N—H···O and intermolecular C—H···O hydrogen bonds stabilizes the packing (Table 1; Fig. 2).

### Experimental

Hexythiazox was synthesized by the reaction of 5-(4-chlorophenyl)-4-methylthiazolidin-2-one and isocyanatocyclohexane in toluene solution in the patent litearture. Crystals suitable for X-ray experiments were obtained by slow evaporation of an ethanol solution.

### Refinement

H atoms bound to C atoms were placed in calculated positions and treated as riding on their parent atoms, with C—H = 0.93 Å (aromatic), C—H = 0.98 Å (methine), C—H = 0.97 Å (methylene), C—H = 0.96 Å (methyl) and with *U*_iso_(H) = 1.2*U*_eq_(C). N-H atoms were initially located in a difference Fourier map but they were treated as riding on their parent atoms with N—H = 0.85 Å, and with *U*_iso_(H) = 1.2*U*_eq_(O).

### Crystal data

**Table:**

C_17_H_21_ClN_2_O_2_S	*F*_000_ = 744
*M**_r_* = 352.88	*D*_x_ = 1.309 Mg m^−^^3^
Monoclinic, *P*2_1_/*c*	Mo *K*α radiation λ = 0.71073 Å
Hall symbol: -P 2ybc	Cell parameters from 12810 reflections
*a* = 10.284 (4) Å	θ = 3.3–27.5º
*b* = 11.799 (5) Å	µ = 0.34 mm^−^^1^
*c* = 15.902 (5) Å	*T* = 291 (2) K
β = 111.830 (14)º	Block, colorless
*V* = 1791.2 (12) Å^3^	0.27 × 0.26 × 0.25 mm
*Z* = 4	

### Data collection

**Table:**

Rigaku R-AXIS RAPID diffractometer	4088 independent reflections
Radiation source: fine-focus sealed tube	3066 reflections with *I* > 2σ(*I*)
Monochromator: graphite	*R*_int_ = 0.033
*T* = 291(2) K	θ_max_ = 27.5º
ω scans	θ_min_ = 3.3º
Absorption correction: multi-scan(ABSCOR; Higashi, 1995)	*h* = −13→11
*T*_min_ = 0.913, *T*_max_ = 0.918	*k* = −15→15
17194 measured reflections	*l* = −19→20

### Refinement

**Table:**

Refinement on *F*^2^	Secondary atom site location: difference Fourier map
Least-squares matrix: full	Hydrogen site location: inferred from neighbouring sites
*R*[*F*^2^ > 2σ(*F*^2^)] = 0.041	H-atom parameters constrained
*wR*(*F*^2^) = 0.109	*w* = 1/[σ^2^(*F*_o_^2^) + (0.0513*P*)^2^ + 0.331*P*] where *P* = (*F*_o_^2^ + 2*F*_c_^2^)/3
*S* = 1.06	(Δ/σ)_max_ = 0.001
4088 reflections	Δρ_max_ = 0.27 e Å^−^^3^
209 parameters	Δρ_min_ = −0.27 e Å^−^^3^
Primary atom site location: structure-invariant direct methods	Extinction correction: none

### Special details

**Table:**

Geometry. All esds (except the esd in the dihedral angle between two l.s. planes) are estimated using the full covariance matrix. The cell esds are taken into account individually in the estimation of esds in distances, angles and torsion angles; correlations between esds in cell parameters are only used when they are defined by crystal symmetry. An approximate (isotropic) treatment of cell esds is used for estimating esds involving l.s. planes.
Refinement. Refinement of F^2^ against ALL reflections. The weighted R-factor wR and goodness of fit S are based on F^2^, conventional R-factors R are based on F, with F set to zero for negative F^2^. The threshold expression of F^2^ > 2sigma(F^2^) is used only for calculating R-factors(gt) etc. and is not relevant to the choice of reflections for refinement. R-factors based on F^2^ are statistically about twice as large as those based on F, and R- factors based on ALL data will be even larger.

### Fractional atomic coordinates and isotropic or equivalent isotropic displacement parameters (Å^2^)

**Table:**

	*x*	*y*	*z*	*U*_iso_*/*U*_eq_	
C1	0.5475 (2)	1.00752 (16)	0.78248 (11)	0.0503 (4)	
C2	0.5027 (2)	1.07700 (16)	0.83577 (12)	0.0564 (5)	
H1	0.5544	1.1405	0.8637	0.068*	
C3	0.3794 (2)	1.05051 (15)	0.84691 (12)	0.0540 (5)	
H2	0.3480	1.0976	0.8823	0.065*	
C4	0.30034 (18)	0.95543 (13)	0.80670 (10)	0.0426 (4)	
C5	0.3492 (2)	0.88779 (15)	0.75323 (11)	0.0509 (4)	
H3	0.2985	0.8239	0.7253	0.061*	
C6	0.4717 (2)	0.91368 (16)	0.74085 (12)	0.0547 (5)	
H4	0.5027	0.8679	0.7045	0.066*	
C7	0.17090 (18)	0.93163 (14)	0.82722 (12)	0.0460 (4)	
H5	0.1281	1.0046	0.8313	0.055*	
C8	0.20030 (17)	0.86779 (12)	0.91720 (11)	0.0392 (4)	
H6	0.2982	0.8802	0.9567	0.047*	
C9	0.1069 (2)	0.90648 (17)	0.96633 (16)	0.0638 (5)	
H7	0.0106	0.9016	0.9262	0.096*	
H8	0.1289	0.9835	0.9859	0.096*	
H9	0.1220	0.8588	1.0181	0.096*	
C10	0.08671 (16)	0.72176 (14)	0.80822 (11)	0.0418 (4)	
C11	0.24351 (16)	0.66650 (13)	0.96508 (10)	0.0382 (3)	
C12	0.27713 (17)	0.46582 (13)	1.00756 (11)	0.0426 (4)	
H11	0.3500	0.4975	1.0615	0.051*	
C13	0.34475 (19)	0.37870 (16)	0.96687 (12)	0.0506 (4)	
H12	0.4216	0.4134	0.9548	0.061*	
H13	0.2767	0.3519	0.9098	0.061*	
C14	0.3994 (2)	0.27909 (16)	1.03110 (15)	0.0640 (6)	
H14	0.4387	0.2227	1.0029	0.077*	
H15	0.4734	0.3049	1.0861	0.077*	
C15	0.2844 (3)	0.22642 (17)	1.05453 (16)	0.0745 (7)	
H16	0.3218	0.1637	1.0959	0.089*	
H17	0.2127	0.1969	1.0000	0.089*	
C16	0.2203 (2)	0.3122 (2)	1.09784 (15)	0.0721 (6)	
H18	0.2902	0.3371	1.1549	0.087*	
H19	0.1441	0.2772	1.1105	0.087*	
C17	0.1650 (2)	0.41410 (18)	1.03619 (13)	0.0561 (5)	
H20	0.0863	0.3908	0.9828	0.067*	
H21	0.1318	0.4707	1.0677	0.067*	
Cl1	0.70303 (6)	1.03996 (6)	0.76763 (4)	0.07627 (19)	
N1	0.18010 (13)	0.74624 (10)	0.89396 (8)	0.0369 (3)	
N2	0.21922 (17)	0.55814 (12)	0.94286 (10)	0.0558 (4)	
H10	0.1648	0.5422	0.8896	0.067*	
O1	0.04055 (13)	0.62916 (10)	0.77874 (8)	0.0545 (3)	
O2	0.31689 (14)	0.70170 (10)	1.03933 (7)	0.0520 (3)	
S1	0.03912 (5)	0.84657 (4)	0.74263 (3)	0.06226 (18)	

### Atomic displacement parameters (Å^2^)

**Table:**

	*U*^11^	*U*^22^	*U*^33^	*U*^12^	*U*^13^	*U*^23^
C1	0.0621 (11)	0.0475 (10)	0.0381 (8)	−0.0070 (8)	0.0149 (8)	0.0067 (7)
C2	0.0721 (12)	0.0442 (10)	0.0500 (10)	−0.0206 (9)	0.0192 (9)	−0.0081 (8)
C3	0.0723 (12)	0.0393 (9)	0.0511 (10)	−0.0112 (8)	0.0238 (9)	−0.0112 (8)
C4	0.0533 (9)	0.0299 (8)	0.0347 (7)	−0.0024 (7)	0.0048 (7)	0.0050 (6)
C5	0.0650 (11)	0.0354 (9)	0.0431 (9)	−0.0083 (8)	0.0092 (8)	−0.0059 (7)
C6	0.0706 (12)	0.0479 (11)	0.0434 (9)	−0.0012 (9)	0.0187 (9)	−0.0042 (8)
C7	0.0457 (9)	0.0291 (8)	0.0518 (9)	0.0032 (7)	0.0047 (7)	0.0046 (7)
C8	0.0427 (8)	0.0263 (7)	0.0462 (8)	−0.0002 (6)	0.0137 (7)	−0.0025 (6)
C9	0.0720 (13)	0.0448 (11)	0.0865 (15)	0.0026 (9)	0.0431 (12)	−0.0134 (10)
C10	0.0359 (8)	0.0384 (9)	0.0428 (8)	0.0002 (6)	0.0049 (7)	0.0003 (7)
C11	0.0430 (8)	0.0312 (8)	0.0389 (8)	−0.0008 (6)	0.0137 (7)	−0.0006 (6)
C12	0.0500 (9)	0.0285 (8)	0.0398 (8)	0.0008 (6)	0.0058 (7)	0.0011 (6)
C13	0.0475 (9)	0.0485 (10)	0.0537 (10)	0.0018 (8)	0.0164 (8)	−0.0029 (8)
C14	0.0599 (12)	0.0448 (11)	0.0698 (12)	0.0186 (9)	0.0039 (10)	−0.0057 (9)
C15	0.0853 (15)	0.0351 (10)	0.0751 (13)	−0.0044 (10)	−0.0026 (12)	0.0133 (9)
C16	0.0732 (13)	0.0748 (15)	0.0641 (12)	−0.0126 (12)	0.0206 (11)	0.0223 (11)
C17	0.0517 (10)	0.0593 (12)	0.0569 (10)	0.0086 (9)	0.0197 (9)	0.0045 (9)
Cl1	0.0787 (4)	0.0850 (4)	0.0734 (3)	−0.0163 (3)	0.0380 (3)	0.0019 (3)
N1	0.0414 (7)	0.0269 (6)	0.0374 (6)	0.0017 (5)	0.0087 (5)	−0.0016 (5)
N2	0.0764 (10)	0.0290 (7)	0.0418 (7)	−0.0011 (7)	−0.0013 (7)	0.0002 (6)
O1	0.0542 (7)	0.0403 (7)	0.0520 (7)	−0.0077 (5)	0.0001 (6)	−0.0066 (5)
O2	0.0719 (8)	0.0352 (6)	0.0370 (6)	−0.0045 (6)	0.0066 (6)	−0.0013 (5)
S1	0.0508 (3)	0.0494 (3)	0.0583 (3)	−0.0045 (2)	−0.0125 (2)	0.0140 (2)

### Geometric parameters (Å, °)

**Table:**

C1—C6	1.374 (3)	C10—S1	1.7655 (18)
C1—C2	1.376 (3)	C11—O2	1.2139 (19)
C1—Cl1	1.744 (2)	C11—N2	1.325 (2)
C2—C3	1.380 (3)	C11—N1	1.4286 (19)
C2—H1	0.9300	C12—N2	1.464 (2)
C3—C4	1.393 (2)	C12—C13	1.514 (2)
C3—H2	0.9300	C12—C17	1.517 (3)
C4—C5	1.389 (3)	C12—H11	0.9800
C4—C7	1.510 (3)	C13—C14	1.521 (3)
C5—C6	1.379 (3)	C13—H12	0.9700
C5—H3	0.9300	C13—H13	0.9700
C6—H4	0.9300	C14—C15	1.501 (3)
C7—C8	1.544 (2)	C14—H14	0.9700
C7—S1	1.8159 (17)	C14—H15	0.9700
C7—H5	0.9800	C15—C16	1.507 (4)
C8—N1	1.4763 (19)	C15—H16	0.9700
C8—C9	1.517 (3)	C15—H17	0.9700
C8—H6	0.9800	C16—C17	1.522 (3)
C9—H7	0.9600	C16—H18	0.9700
C9—H8	0.9600	C16—H19	0.9700
C9—H9	0.9600	C17—H20	0.9700
C10—O1	1.214 (2)	C17—H21	0.9700
C10—N1	1.375 (2)	N2—H10	0.8445
			
C6—C1—C2	121.10 (19)	N2—C12—C17	110.75 (15)
C6—C1—Cl1	119.77 (16)	C13—C12—C17	112.11 (15)
C2—C1—Cl1	119.14 (15)	N2—C12—H11	108.1
C1—C2—C3	118.59 (17)	C13—C12—H11	108.1
C1—C2—H1	120.7	C17—C12—H11	108.1
C3—C2—H1	120.7	C12—C13—C14	110.57 (16)
C2—C3—C4	122.04 (18)	C12—C13—H12	109.5
C2—C3—H2	119.0	C14—C13—H12	109.5
C4—C3—H2	119.0	C12—C13—H13	109.5
C5—C4—C3	117.47 (18)	C14—C13—H13	109.5
C5—C4—C7	125.03 (15)	H12—C13—H13	108.1
C3—C4—C7	117.46 (16)	C15—C14—C13	110.99 (16)
C6—C5—C4	121.16 (16)	C15—C14—H14	109.4
C6—C5—H3	119.4	C13—C14—H14	109.4
C4—C5—H3	119.4	C15—C14—H15	109.4
C1—C6—C5	119.64 (18)	C13—C14—H15	109.4
C1—C6—H4	120.2	H14—C14—H15	108.0
C5—C6—H4	120.2	C14—C15—C16	110.77 (17)
C4—C7—C8	113.97 (13)	C14—C15—H16	109.5
C4—C7—S1	114.69 (13)	C16—C15—H16	109.5
C8—C7—S1	104.59 (11)	C14—C15—H17	109.5
C4—C7—H5	107.8	C16—C15—H17	109.5
C8—C7—H5	107.8	H16—C15—H17	108.1
S1—C7—H5	107.8	C15—C16—C17	111.00 (18)
N1—C8—C9	111.37 (14)	C15—C16—H18	109.4
N1—C8—C7	106.33 (13)	C17—C16—H18	109.4
C9—C8—C7	112.78 (15)	C15—C16—H19	109.4
N1—C8—H6	108.8	C17—C16—H19	109.4
C9—C8—H6	108.8	H18—C16—H19	108.0
C7—C8—H6	108.8	C12—C17—C16	111.58 (16)
C8—C9—H7	109.5	C12—C17—H20	109.3
C8—C9—H8	109.5	C16—C17—H20	109.3
H7—C9—H8	109.5	C12—C17—H21	109.3
C8—C9—H9	109.5	C16—C17—H21	109.3
H7—C9—H9	109.5	H20—C17—H21	108.0
H8—C9—H9	109.5	C10—N1—C11	126.26 (13)
O1—C10—N1	126.92 (15)	C10—N1—C8	115.70 (12)
O1—C10—S1	122.68 (12)	C11—N1—C8	117.47 (12)
N1—C10—S1	110.40 (11)	C11—N2—C12	122.90 (14)
O2—C11—N2	125.23 (15)	C11—N2—H10	118.1
O2—C11—N1	118.71 (14)	C12—N2—H10	118.9
N2—C11—N1	116.04 (13)	C10—S1—C7	93.26 (8)
N2—C12—C13	109.64 (15)		

### Hydrogen-bond geometry (Å, °)

**Table:**

*D*—H···*A*	*D*—H	H···*A*	*D*···*A*	*D*—H···*A*
N2—H10···O1	0.84	2.03	2.706 (2)	137
C2—H1···O2^i^	0.93	2.47	3.386 (2)	170
C5—H3···S1	0.93	2.79	3.168 (2)	105
C12—H11···O2	0.98	2.44	2.831 (2)	103

Symmetry codes: (i) −*x*+1, −*y*+2, −*z*+2.
